# Supplementary material for: Resolving discrepancies between chimeric and multiplicative measures of higher-order epistasis
Source: Nat Commun. 2025 Feb 17;16:1711. doi: 10.1038/s41467-025-56986-5 (PMC11833126; doi:10.1038/s41467-025-56986-5)
Supplement: Supplementary file 1 — Supplementary Information [file 41467_2025_56986_MOESM1_ESM.pdf]

Supplementary information for “Resolving discrepancies between chimeric and multiplicative measures of higher-order epistasis”

Uthsav Chitra*[1], Brian Arnold*[1,2], and Benjamin J. Raphael†[1]

[1]Department of Computer Science, Princeton University, Princeton, NJ, USA
[2]Center for Statistics and Machine Learning, Princeton University, Princeton, NJ, USA

*These authors contributed equally.
†Correspondence: braphael@princeton.edu

Supplementary Note 1 Pairwise and higher-order epistasis comparison

The pairwise chimeric measure ϵ_{ij}^C approximates the pairwise multiplicative epistasis measure ϵ_{ij}^M under certain conditions. Specifically, if the double mutant fitness f_{ij} and the product $f_i f_j$ of the single-mutant fitness values are both close to 1, i.e. $f_{ij} \approx 1$ and $f_i f_j \approx 1$, then the pairwise chimeric epistasis measure ϵ_{ij}^C is approximately equal to the pairwise log-multiplicative measure $\log \epsilon_{ij}^M$. To see this, note that

$$\log \epsilon_{ij}^M = \log f_{ij} - \log f_i f_j \approx (f_{ij} - 1) - (f_i f_j - 1) = f_{ij} - f_i f_j = \epsilon_{ij}^C, \tag{1}$$

where we use the approximation that $\log c \approx c - 1$ if $c \approx 1$.

We empirically assessed (Supplementary Figure 1) how closely the the pairwise chimeric epistasis measure ϵ_{ij}^C approximates the interaction parameter β_{ij} (which is equal to the pairwise log-multiplicative measure $\log \epsilon_{ij}^M$), using the simulated fitness values $\mathbf{f}$ from the multiplicative fitness model (Section 2.4, pairwise interactions $K = 2$ with noise parameter $\sigma = 0$). We observe that the chimeric measure has small error $|\epsilon_{ij}^C - \beta_{ij}|$ when both the double mutant fitness f_{ij} and product $f_i f_j$ of single mutant fitness values are close to 1. However, the error gets much larger when either f_{ij} or $f_i f_j$ are not close to 1, which agrees with when the the approximation in (1) is valid. Moreover, the multiplicative and chimeric measures diverge substantially for higher-order interactions, with the correlation between the two measures approaching zero as the interaction order increases (Supplementary Figure 2).

This analysis demonstrates that for pairwise interactions, the chimeric epistasis measure ϵ_{ij}^C is an accurate measure of interactions in a multiplicative fitness model in certain settings.

Supplementary Note 2 Comparing chimeric and multiplicative formulae

For pairwise epistasis, the sign of the chimeric measure ϵ^C is always equal to the sign of the multiplicative epistasis measure ϵ^M (Proposition 1). Nevertheless, the chimeric epistasis measure may sometimes overstate or understate the degree of epistasis, as we demonstrate below.

Example 1. *Consider the two following scenarios under a multiplicative fitness model:*

1. $f_{11} = 0.8, f_{10} = f_{01} = 1$, *versus*
2. $f_{11} = 0.2, f_{10} = f_{01} = 0.5$.

In both scenarios, the multiplicative epistasis measure is given by $\epsilon^M = 0.8 < 1$, *indicating the same degree of negative epistasis. However, the chimeric epistasis measure* ϵ^C *gives a different conclusion, namely that the first scenario (*$\epsilon^C = -0.2 < 0$*) has a larger degree of negative epistasis compared to the second scenario (*$\epsilon^C = -0.05 < 0$*).*

For higher-order epistasis, the sign of the chimeric epistasis measure is not guaranteed to be equal to the sign of the multiplicative epistasis measure, as we demonstrate below.

Example 2. *Consider a genotype with* $L = 3$ *loci and the following fitness values under the multiplicative model:*

- $f_1 = 0.5, f_2 = 1.0, f_3 = 0.25$,
- $f_{12} = 0.5$, $f_{13} = 1$, $f_{23} = 0.1$,
- $f_{123} = 0.4$

Then there is no three-way epistasis using the multiplicative epistasis formula, i.e.,

$$\epsilon_{123}^M = 1 \tag{2}$$

However the chimeric three-way epistasis measure ϵ_{123}^C *is given by*

$$\epsilon_{123}^C = -0.525 < 0 \tag{3}$$

and would indicate a negative three-way interaction.

If instead we have $f_1 = 0.1$ *and* $f_{123} = 2$ *then there is still no three-way epistasis according to the multiplicative measure, i.e.* $\epsilon_{123}^M = 1$, *but the chimeric three-way measure would incorrectly indicate a* positive *three-way interaction with* $\epsilon_{123}^C = 0.915$.

Supplementary Note 3 Mathematical results

3.1 Proof of Proposition 1

Proposition 1. *Let $f_{00}, f_{01}, f_{10}, f_{11} \in \mathbb{R}$ be real numbers. Let $\epsilon^M = \frac{f_{11}f_{00}}{f_{01}f_{10}}$ and $\epsilon^C = f_{11} - \frac{f_{01}f_{10}}{f_{00}}$. Then*

$$\operatorname{sgn}(\epsilon^C) = \operatorname{sgn}(\epsilon^M - 1). \tag{4}$$

Proof. We have that

$$\epsilon^C > 0 \Longleftrightarrow f_{11} - \frac{f_{01}f_{10}}{f_{00}} > 0 \Longleftrightarrow \frac{f_{11}f_{00}}{f_{01}f_{10}} > 1 \Longleftrightarrow \epsilon^M - 1 > 0. \tag{5}$$

Thus, $\epsilon^C > 0$ if and only if $\epsilon^M - 1 > 0$. By similar logic, we have that $\epsilon^C = 0$ (resp. $\epsilon^C < 0$) if and only if $\epsilon^M - 1 > 0$ (resp. $\epsilon^M - 1 < 0$). It follows that $\operatorname{sgn}(\epsilon^C) = \operatorname{sgn}(\epsilon^M - 1)$. □

3.2 Proof of Theorem 1

Theorem 1. *Let $(X_1, \ldots, X_L) \in \{0,1\}^L$ follow the multivariate Bernoulli distribution with general parameters $\mathbf{p}$ and natural parameters $\boldsymbol{\beta}$. Let $f_{x_1 \cdots x_L} \in \mathbb{R}$ be real numbers for each $(x_1, \cdots, x_L) \in \{0,1\}^L$ such that $f_{x_1 \cdots x_L} = c \cdot p_{x_1 \ldots x_L}$ for some constant $c \in \mathbb{R}$. Then we have*

$$\beta_S = \log \epsilon_S^M. \tag{6}$$

Proof. We proceed by induction on the size $|S|$ of S. For our base case we assume that $|S| = 2$. Writing $S = \{i, j\}$, then we have that

$$\begin{aligned} \epsilon_{ij}^M &= \frac{f_{ij} f_\varnothing}{f_i f_j} \\ &= \frac{p_{ij} p_\varnothing}{p_i p_j} \\ &= \frac{\exp(\beta_\varnothing + \beta_1 + \beta_2 + \beta_{12} \exp(\beta_\varnothing)}{\exp(\beta_{\beta_\varnothing} + \beta_1) \exp(\beta_{\beta_\varnothing} + \beta_2)} \\ &= \exp(\beta_{12}), \end{aligned} \tag{7}$$

where in the second equality we use that the fitness values f are proportional to the probabilities p of the multivariate Bernoulli, and in the third equality we use the definition (23) of the natural parameters $\boldsymbol{\beta}$.

Next for the inductive hypothesis we assume that $\beta_S = \log \epsilon_S^M$ holds for all $|S| < K$. We will prove the equation holds for $|S| = K$. Without loss of generality assume that $S = \{1, \ldots, K\}$. Then from (18) we have

$$\begin{aligned} \epsilon_{1 \cdots k}^M &= \frac{f_{1 \ldots K}}{f_\varnothing \left(\prod_{i=1}^K \frac{f_i}{f_\varnothing} \right) \left(\prod_{1 \le i_1 < i_2 \le K} \epsilon_{i_1 i_2}^M \right) \cdots \left(\prod_{1 \le i_1 < \cdots < i_{K-1} \le K} \epsilon_{i_1 \cdots i_{K-1}}^M \right)} \\ &= \frac{p_{1 \ldots K}}{p_\varnothing \left(\prod_{i=1}^K \frac{p_i}{p_\varnothing} \right) \left(\prod_{1 \le i_1 < i_2 \le K} \epsilon_{i_1 i_2}^M \right) \cdots \left(\prod_{1 \le i_1 < \cdots < i_{K-1} \le K} \epsilon_{i_1 \cdots i_{K-1}}^M \right)} \\ &= \frac{\exp\left(\beta_\varnothing + \sum_{i=1}^K \beta_i + \sum_{1 \le i_1 < i_2 \le K} \beta_{i_1 i_2} + \cdots + \sum_{1 \le i_1 < \cdots < i_{K-1} \le K} \beta_{i_1 \cdots i_{K-1}} + \beta_{1 \cdots k} \right)}{\exp(\beta_\varnothing) \left(\prod_{i=1}^K \exp(\beta_i) \right) \left(\prod_{1 \le i_1 < i_2 \le K} \exp(\beta_{i_1 i_2}) \right) \cdots \left(\prod_{1 \le i_1 < \cdots < i_{K-1} \le K} \exp(\beta_{i_1 \cdots i_{K-1}}^M) \right)} \\ &= \exp(\beta_{1 \cdots K}), \end{aligned} \tag{8}$$

which completes the proof. □

3.3 Transformation between parameterizations of the multivariate Bernoulli

Let $(X_1, \dots, X_n) \in \{0,1\}^n$ be distributed according to a multivariate Bernoulli distribution with natural parameters $\boldsymbol{\beta}$, mean parameters $\boldsymbol{\mu}$, and general parameters $\mathbf{p}$. [1, 2] give two formulae relating these different parametrizations. These equations are defined in terms of the *Kronecker product* $A \otimes B$ of matrices A, B. For shorthand, we write $A^{\otimes n} = A \otimes A \cdots \otimes A$ as the Kronecker product of a matrix A with itself n times. Note that if A has size $s \times t$ then $A^{\otimes n}$ has size $s^n \times t^n$.

First, Equation (13) of [1] gives the following formula relating the natural parameters $\boldsymbol{\beta}$ and the general parameters $\mathbf{p}$:

$$\log \mathbf{p} = \begin{pmatrix} 1 & 0 \\ 1 & 1 \end{pmatrix}^{\otimes n} \boldsymbol{\beta} \tag{9}$$

where $\log$ is taken entry-wise. Note that $\begin{pmatrix} 1 & 0 \\ 1 & 1 \end{pmatrix}^{\otimes n}$ is a square matrix of size $2^n \times 2^n$.

Second, [2] gives the following formula relating the general parameters $\mathbf{p}$ and the mean parameters $\boldsymbol{\mu}$:

$$\boldsymbol{\mu} = \begin{pmatrix} 1 & 1 \\ 0 & 1 \end{pmatrix}^{\otimes n} \mathbf{p}. \tag{10}$$

3.4 Proof of Theorem 2

We require the following lemma.

Lemma 1. *Let $(X_1, \dots, X_L) \in \{0,1\}^L$ be distributed according to a multivariate Bernoulli distribution with moments $\boldsymbol{\mu}^C$. Define $Y_\ell = 1 - 2X_\ell \in \{-1, 1\}$ for $\ell = 1, \cdots, L$. Let $\boldsymbol{\mu}^Y = [\mu^Y_{x_1 \cdots x_L}] \in \mathbb{R}^{2^L}$ be a vector with entries $u_{x_1 \cdots x_L} = E[Y_1^{x_1} \cdots Y_L^{x_L}]$. Then we have*

$$\boldsymbol{\mu}^Y = \begin{pmatrix} 1 & 0 \\ 1 & -2 \end{pmatrix}^{\otimes L} \boldsymbol{\mu}^C. \tag{11}$$

Proof. We proceed by induction on L. For ease of notation, we define $M_\ell = \begin{pmatrix} 1 & 0 \\ 1 & -2 \end{pmatrix}^{\otimes \ell}$. The base case, $L = 1$, is equivalent to

$$\begin{pmatrix} 1 \\ 1 - 2 \cdot E[X_1] \end{pmatrix} = \begin{pmatrix} 1 & 0 \\ 1 & -2 \end{pmatrix} \begin{pmatrix} 1 \\ E[X_1] \end{pmatrix}, \tag{12}$$

which holds by inspection.

Now for the inductive step, we assume (11) holds for $L - 1$ and we will show (11) holds for L. Define $A = \{(a_1, \cdots, a_L \in \{0,1\}^L : a_1 = 0\}$ and $B = \{(a_1, \cdots, a_L \in \{0,1\}^L : a_1 = 1\}$. Then the first 2^{L-1} entries of $\boldsymbol{\mu}^Y, \boldsymbol{\mu}^C$ are indexed by A, and the second 2^{L-1} entries are indexed by B. Thus, define $\boldsymbol{\mu}^Y_A, \boldsymbol{\mu}^C_A$ as the first 2^{L-1} entries of $\boldsymbol{\mu}^Y, \boldsymbol{\mu}^C$, respectively, and define $\boldsymbol{\mu}^Y_B, \boldsymbol{\mu}^C_B$ similarly.

For $(0, a_2, \cdots, a_L) \in A$, we have that

$$(\boldsymbol{\mu}^Y_A)_{0a_2 \cdots a_L} = E[Y_1^0 Y_2^{a_2} \cdots Y_L^{a_L}] = E[Y_2^{a_2} \cdots Y_L^{a_L}] = (M_{L-1} \boldsymbol{\mu}^C_A)_{0a_2 \cdots a_L}, \tag{13}$$

where in the last equality we use the inductive hypothesis on the $L - 1$ random variables $(X_2, \cdots, X_L)$. Similarly for $(1, a_2, \cdots, a_L) \in B$, we have

$$\begin{aligned} (\boldsymbol{\mu}^Y_B)_{1a_2 \cdots a_L} &= E[Y_1^1 Y_2^{a_2} \cdots Y_L^{a_L}] \\ &= E[(1 - 2X_1) Y_2^{a_2} \cdots Y_L^{a_L}] \\ &= E[Y_2^{a_2} \cdots Y_L^{a_L}] - 2 \cdot E[X_1 Y_2^{a_2} \cdots Y_L^{a_L}] \\ &= (M_{L-1} \boldsymbol{\mu}^C_A)_{0a_2 \cdots a_L} - 2(M_{L-1} \boldsymbol{\mu}^C_B)_{1a_2 \cdots a_L}, \end{aligned} \tag{14}$$

where again in the last equality we use the inductive hypothesis. Writing these two equalities in matrix form:

$$\left(\begin{array}{c}\boldsymbol{\mu}_A^Y \\ \hline \boldsymbol{\mu}_B^Y\end{array}\right) = \left(\begin{array}{c|c} M_{L-1} & 0 \\ \hline M_{L-1} & -2M_{L-1}\end{array}\right)\left(\begin{array}{c}\boldsymbol{\mu}_A^C \\ \hline \boldsymbol{\mu}_B^C\end{array}\right). \quad (15)$$

By definition of the Kronecker product, the matrix in (15) is equal to

$$\left(\begin{array}{c|c} M_{L-1} & 0 \\ \hline M_{L-1} & -2M_{L-1}\end{array}\right) = M_{L-1} \otimes \begin{pmatrix}1 & 0 \\ 1 & -2\end{pmatrix} = M_{L-1} \otimes M_1 = M_L. \quad (16)$$

Thus, $\boldsymbol{\mu}^Y = M_L \boldsymbol{\mu}^C$, completing the proof. □

Theorem 2. *Let* $(X_1, \ldots, X_L) \in \{0,1\}^L$ *be distributed according to a multivariate Bernoulli distribution with general parameters* $\mathbf{f}$*, and define* $Y_\ell = 1 - 2X_\ell \in \{-1, 1\}$*. Define* $\mathbf{u} = [u_{x_1 \cdots x_L}] \in \mathbb{R}^{2^L}$ *as in* (32)*. Then* $u_{x_1 \cdots x_L} = E[Y_1^{x_1} \cdots Y_L^{x_L}]$.

Proof. Let $\boldsymbol{\mu}^C$ be the mean parameters of X. We define the vector $\boldsymbol{\mu}^Y = [\mu^Y_{x_1 \cdots x_L}] \in \mathbb{R}^{2^L}$ with entries $\mu^Y_{x_1 \cdots x_L} = E[Y_1^{x_1} \cdots Y_L^{x_L}]$. By Lemma 1 and equation (10), we have

$$\boldsymbol{\mu}^Y = \begin{pmatrix}1 & 0 \\ 1 & -2\end{pmatrix}^{\otimes L} \boldsymbol{\mu}^C = \begin{pmatrix}1 & 0 \\ 1 & -2\end{pmatrix}^{\otimes L} \begin{pmatrix}1 & 1 \\ 0 & 1\end{pmatrix}^{\otimes L} \mathbf{f} = \left(\begin{pmatrix}1 & 0 \\ 1 & -2\end{pmatrix} \cdot \begin{pmatrix}1 & 1 \\ 0 & 1\end{pmatrix}\right)^{\otimes L} \mathbf{f} = \begin{pmatrix}1 & 1 \\ 1 & -1\end{pmatrix}^{\otimes L} \mathbf{f}. \quad \square$$

Supplementary Note 4 Yeast reanalysis details

4.1 Reproducing previous results

We gathered fitness data from the supplemental tables of [3] and [4] in a way that was consistent with how the data were described in the corresponding publications. To reproduce results from [3], we used data from Supplementary Tables S1, S3, and S5. To reproduce those from [4] we used Table S1 along with Data File S1 from [5] which contains three tables (SGA_NxN.txt, SGA_ExN.txt, SGA_ExE.txt).

Following the formula and notation from the supplement of [4], we recalculated the trigenic interaction scores τ_{ijk} reported by [4, 3] (i.e. the chimeric three-way measures ϵ^C_{ijk}) using the following formula:

$$\tau_{ijk} = e_{ij,k} - e_{ik} f_j - e_{jk} f_i \quad (17)$$

where

$$e_{ij,k} = f_{ijk} - f_{ij} f_k. \quad (18)$$

Here, e_{jk} and e_{ik} are the reported pairwise interactions between knockout mutant j, k and i, k, respectively. We obtained the values of $f_i, f_j, f_k, f_{ij}, f_{ijk}, e_{ik}$, and e_{jk} directly from the data tables of [4, 3]. We recomputed the trigenic interaction score τ_{ijk} for 74% and 81% of the triple knockout mutants (i, j, k) reported in [3] and [4], respectively, as some values were either missing or were "NaN". Our recalculated trigenic interaction scores were highly similar to reported values: the distributions were nearly identical and values were highly correlated (Supplementary Figure 3), with a Pearson correlation of 0.9974 and 0.9809 for the data from [3] and [4], respectively.

We note that plugging (18) into (17) yields the following expression for the trigenic interaction score τ_{ijk}:

$$\tau_{ijk} = f_{ijk} + 2 f_i f_j f_k - f_i f_{jk} - f_j f_{ik} - f_k f_{ij}. \quad (19)$$

Computing the trigenic interaction score τ_{ijk} using (19) should be identical to computing it using (17) (Supplementary Figure 3). However, we find that the scores calculated using (19) are quite different compared to the scores reported by [3, 4] (Supplementary Figure 4). This discrepancy is due to the fact that the reported values of f_{ik} and f_{jk} are not equal to $f_i f_k + e_{ik}$ and $f_j f_k + e_{jk}$, respectively. Thus, to make our reanalysis consistent with the reported trigenic interaction scores from [4, 3], we recompute the trigenic interaction score using (17).

We computed the three-way multiplicative measure ϵ^M_{ijk} as

$$\epsilon^M_{ijk} = \frac{f_{ijk} f_i f_j f_k}{f_{ij}(f_i f_k + e_{ik})(f_j f_k + e_{jk})}. \quad (20)$$

In the denominator, we compute the double-mutant fitness values f_{ik} and f_{jk} indirectly due to the aforementioned issues with using the reported double-mutant fitness values f_{ik} and f_{jk}. Our data handling procedures and analysis are located in our Github repository.

4.2 Enrichment analyses

Physical interactions. To test whether subsets of trigenic interactions were enriched for protein-protein interactions, we downloaded pairwise physical interaction data from BIOGRID (release 4.4.211) for Saccharomyces cerevisiae (strain S288c) and only considered physical interactions discovered using the following experimental system types: Affinity Capture-MS, Affinity Capture-Western, Two-hybrid, Reconstituted Complex, PCA, Co-purification, and Co-crystal Structure. Using this information, we classified a given set of three genes as having a shared protein-protein interaction if all three genes had a physical interaction with at least one other gene in the genome (not including the genes in the set of three).

Coexpression. From COXPRESSdb [6], we downloaded union-type coexpression data, which integrates RNAseq and microarray coexpression data, for S. cerevisiae. From these data, we only considered a pair of genes as significantly coexpressed if they had a Z-score greater than or equal to 3. To test whether subsets of trigenic interactions were enriched for coexpressed genes, we classified a given set of three genes as coexpressed if at least two of three possible gene pairs within the set had a coexpression Z-score greater than or equal to three.

GO enrichment. We downloaded GO Slim mappings from the Saccharomyces Genome Database and only considered GO terms corresponding to biological processes (i.e. with a GO aspect of "P" in the GO_Aspect column of go_slim_mapping.tab). We then classified a given set of three genes as sharing a GO term if all three genes had at least one GO term in common.

4.3 Trigenic interaction fraction

Kuzmin et al. [3] quantify functional redundancy between paralogs using a quantity that they call the *trigenic interaction fraction*. The trigenic interaction fraction is equal to the number of trigenic interactions involving both paralogs divided by the sum of numerator and the number of digenic interactions involving at least one of the paralogs. Kuzmin et al. [3] hypothesize that paralogs with higher trigenic interaction fractions are more redundant, whereas those with low trigenic fractions have undergone subfunctionalization.

We compared the trigenic interaction fraction computed using the chimeric formula to the trigenic interaction fraction computed using the multiplicative formula (Supplementary Figure 7). We observe that for most gene pairs, the trigenic interaction fraction is larger when it is computed using the multiplicative formula versus when it is computed using the chimeric formula. Additionally, for the 15 paralogs that have many additional trigenic interactions found using the multiplicative formula (highlighted in Figures 4C, D), we also observe elevated trigenic interaction fractions (Supplementary Figure 7). This observation further supports the conclusion that the multiplicative measure uncovers additional functional redundancies between these paralogs.

Supplementary Note 5 Relating MVB and case-control GWAS

Suppose we are given genotype $(X_1, X_2) \in \{0,1\}^2$ and (binary) disease status $D \in \{0,1\}$. Then the joint random variable (X_1, X_2, D) follows a MVB distribution, where the log-probability $\log P(X_1, X_2, D)$ is given by the following expression in terms of the natural parameters $\boldsymbol{\beta}$:

$$\log P(X_1, X_2, D) = \beta_0 + \beta_1 X_1 + \beta_2 X_2 + \beta_d D + \beta_{12} X_1 X_2 + \beta_{1d} X_1 D + \beta_{2d} X_2 D + \beta_{12d} X_1 X_2 D. \quad (21)$$

Logistic regression. In the logistic regression approach for measuring pairwise interactions, one fits a model of the form

$$\log\left(\frac{P(D=1|X_1=x_1, X_2=x_2)}{P(D=0|X_1=x_1, X_2=x_2)}\right) = \alpha_0 + \alpha_1 x_1 + \alpha_2 x_2 + \alpha_{12} x_1 x_2 \quad (22)$$

and measures pairwise interactions with the interaction term α_{12}.

To relate the MVB and logistic regression, we rewrite the LHS of 22 as $\log P(X_1 = x_1, X_2 = x_2, D = 1)) - \log P(X_1 = x_1, X_2 = x_2, D = 0)$ and plug in 21:

$$\begin{aligned}
\log\left(\frac{P(D=1|X_1=x_1,X_2=x_2)}{P(D=0|X_1=x_1,X_2=x_2)}\right) &= \log\left(\frac{P(X_1=x_1,X_2=x_2,D=1)\cdot P(X_1=x_1,X_2=x_2)}{P(X_1=x_1,X_2=x_2,D=0)\cdot P(X_1=x_1,X_2=x_2)}\right)\\
&= \log\left(\frac{P(X_1=x_1,X_2=x_2,D=1)}{P(X_1=x_1,X_2=x_2,D=0)}\right)\\
&= \log P(X_1=x_1,X_2=x_2,D=1)) - \log P(X_1=x_1,X_2=x_2,D=0)\\
&= (\beta_0+\beta_1x_1+\beta_2x_2+\beta_d+\beta_{12}x_1x_2+\beta_{1d}x_1+\beta_{2d}x_2+\beta_{12d}x_1x_2)\\
&\quad -(\beta_0+\beta_1x_1+\beta_2x_2+\beta_{12}x_1x_2)\\
&= \beta_d+\beta_{1d}x_1+\beta_{2d}x_2+\beta_{12d}x_1x_2.
\end{aligned} \tag{23}$$

By equating coefficients with (22), it follows that $\alpha_{12} = \beta_{12d}$. That is, the 3-way interaction term β_{12d} in the MVB is equal to the logistic regression interaction term α_{12}.

Conditional independence testing. We start by describing the conditional independence test. Let $\theta_0 = \log\left(\frac{p_{000}p_{110}}{p_{100}p_{010}}\right)$ be the log-odds ratio of X_1 and X_2 conditioned on $D = 0$, where we use $p_{x_1x_2d}$ as shorthand for $P(X = x_1, X_2 = x_2, D = d)$. Note that $\theta_0 = 0$ if and only if X_1 and X_2 are independent conditioned on $D = 0$. Similarly, we define $\theta_1 = \log\left(\frac{p_{001}p_{111}}{p_{101}p_{011}}\right)$ as the log-odds ratio of X_1 and X_2 conditioned on $D = 1$, so that $\theta_1 = 0$ if and only if X_1 and X_2 are independent conditioned on $D = 1$.

The conditional independence test is testing the null hypothesis $H_0 : \theta_0 = \theta_1 = 0$. To relate the conditional independence test to the MVB, we plug (21) into the formula for θ_0 and simplify, which yields:

$$\begin{aligned}
\theta_0 &= \log\left(\frac{p_{000}p_{110}}{p_{100}p_{010}}\right)\\
&= \log p_{000} + \log p_{110} - \log p_{100} - \log p_{010}\\
&= (\beta_0) + (\beta_0+\beta_1+\beta_2+\beta_{12}) - (\beta_0+\beta_1) - (\beta_0+\beta_2)\\
&= \beta_{12}.
\end{aligned} \tag{24}$$

A similar computation for the formula for θ_1 yields

$$\theta_1 = \beta_{12} + \beta_{12d}. \tag{25}$$

Thus, the conditional independence null hypothesis $H_0 : \theta_0 = \theta_1 = 0$ is equivalent to the null hypothesis

$$H_0 : \beta_{12} = \beta_{12d} = 0. \tag{26}$$

In other words, the conditional independence test is testing whether the 2-way interaction β_{12} (i.e. the *marginal* interaction) and 3-way interaction term β_{12d} are equal to 0.

Computing interaction terms β from GWAS data. From Theorem 1, each MVB interaction term is equal to the corresponding log-multiplicative measure, i.e. $\beta = \log \epsilon^M$, where the fitness values $f_{x_1x_2d}$ in the definition of the epistasis measure ϵ^M are proportional to the genotype probability $p_{x_1x_2d} = P(X_1 = x_1, X_2 = x_2, D = d)$, i.e. $f_{x_1x_2d} = c \cdot p_{x_1x_2d}$ for some $c > 0$. Given observational GWAS data, the genotype probability $p_{x_1x_2d}$ – and thus the fitness values – are estimated by the empirical frequency of genotypes in the data with loci $X_1 = x_1, X_2 = x_2$ and disease status $D = d$.

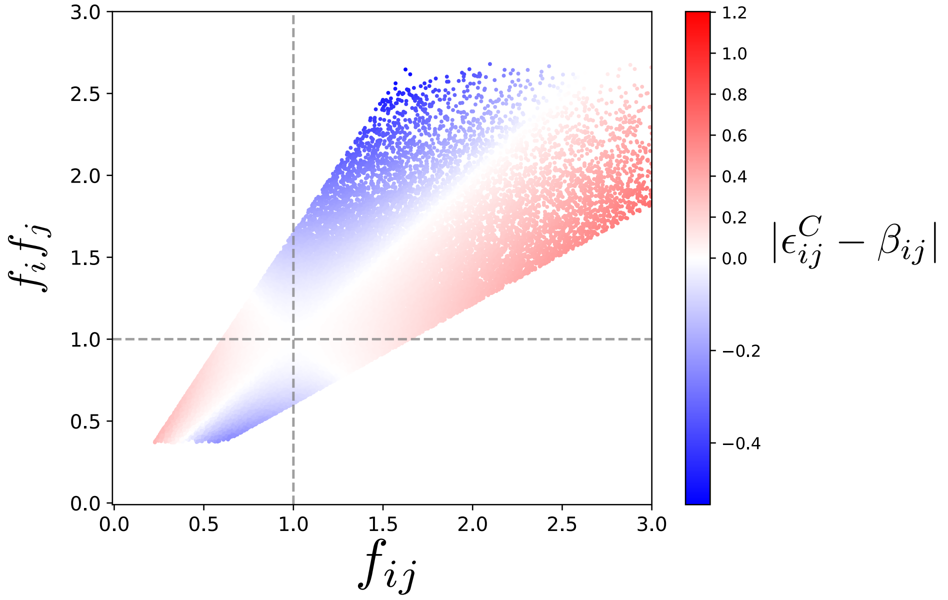

Supplementary Figure 1: **Difference between chimeric and multiplicative epistasis measures for a range of double- and single-mutant fitness values.** Double mutant fitness f_{ij} versus product $f_i f_j$ of single mutant fitness values for each pair (i, j) of loci across all simulated instances of the multiplicative fitness model in Section 2.4 (pairwise interactions with noise parameter $\sigma = 0$). Points are colored by the difference $|\epsilon_{ij}^C - \beta|$ between the chimeric epistasis measure ϵ_{ij}^C and true interaction parameter β.

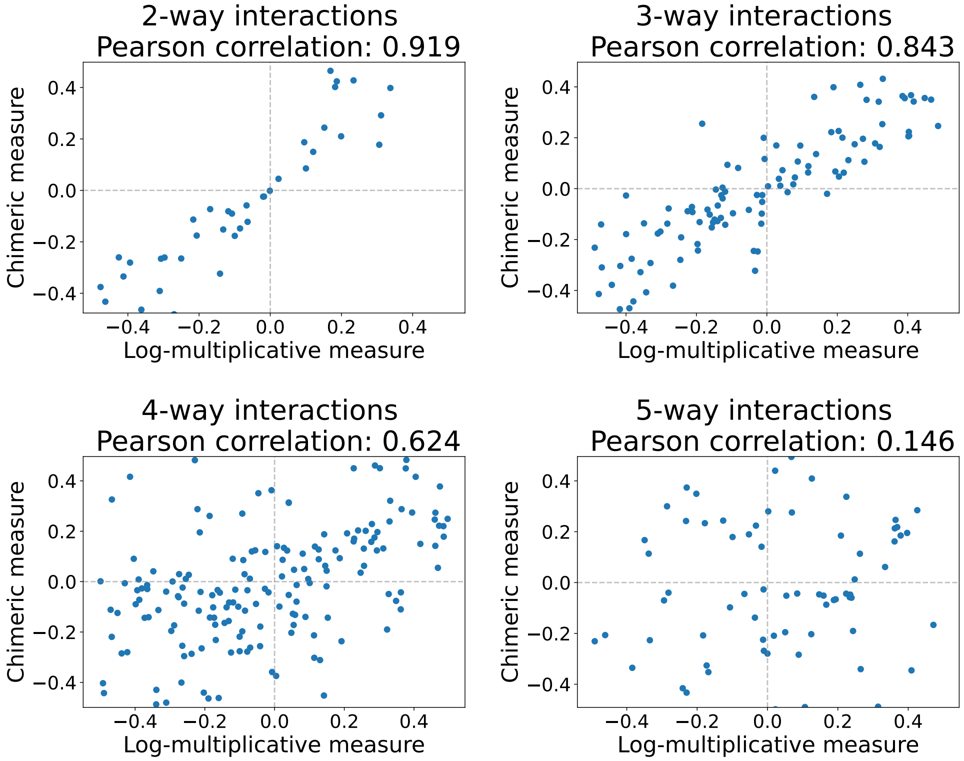

Supplementary Figure 2: **Pearson correlation between chimeric and log-multiplicative epistasis measures for pairwise and higher-order interactions on simulated data.** Pearson correlation $\rho(\epsilon^C, \log \epsilon^M)$ between chimeric measure ϵ^C and multiplicative measure ϵ^M for different interaction orders K and each K-tuple of loci across all simulated instances of the multiplicative fitness model in Section 2.4 (pairwise interactions with noise parameter $\sigma = 0$). The x- and y-axis ranges are $[\min \epsilon^M, \max \epsilon^M]$.

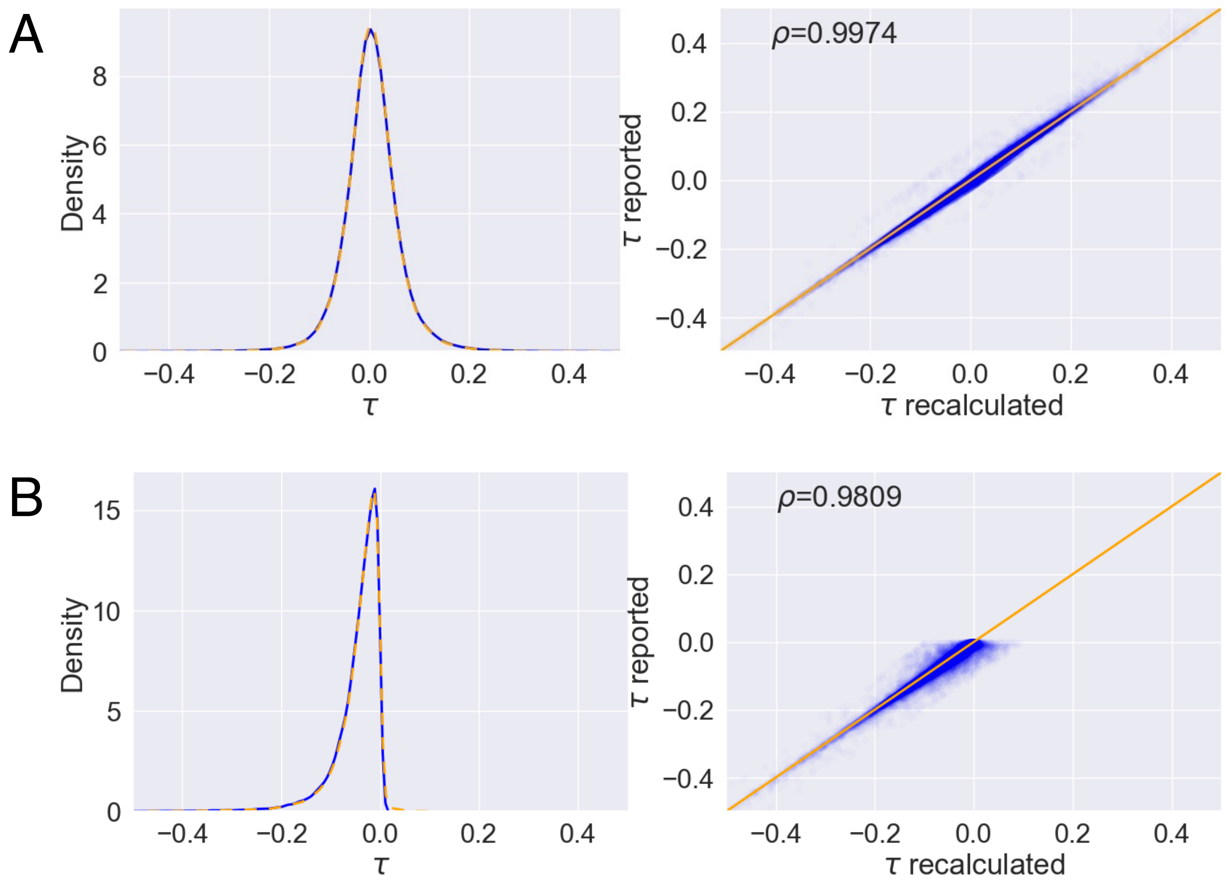

Supplementary Figure 3: **Replication of trigenic interaction scores from Kuzmin et al. [4, 3].** **(A)** Kuzmin et al. (2020) [3]. Left: Density of the reported trigenic interaction score τ_{ijk} (blue) and our recalculation of τ_{ijk} using (17) from fitness data (orange). Right: Calculation of the Pearson correlation between the reported value of τ_{ijk} and our recalculation of τ_{ijk} using (17). **(B)** Same analysis as in (A) for data from Kuzmin et al. (2018) [4]. Note that for this analysis, only negative values of τ_{ijk} were reported.

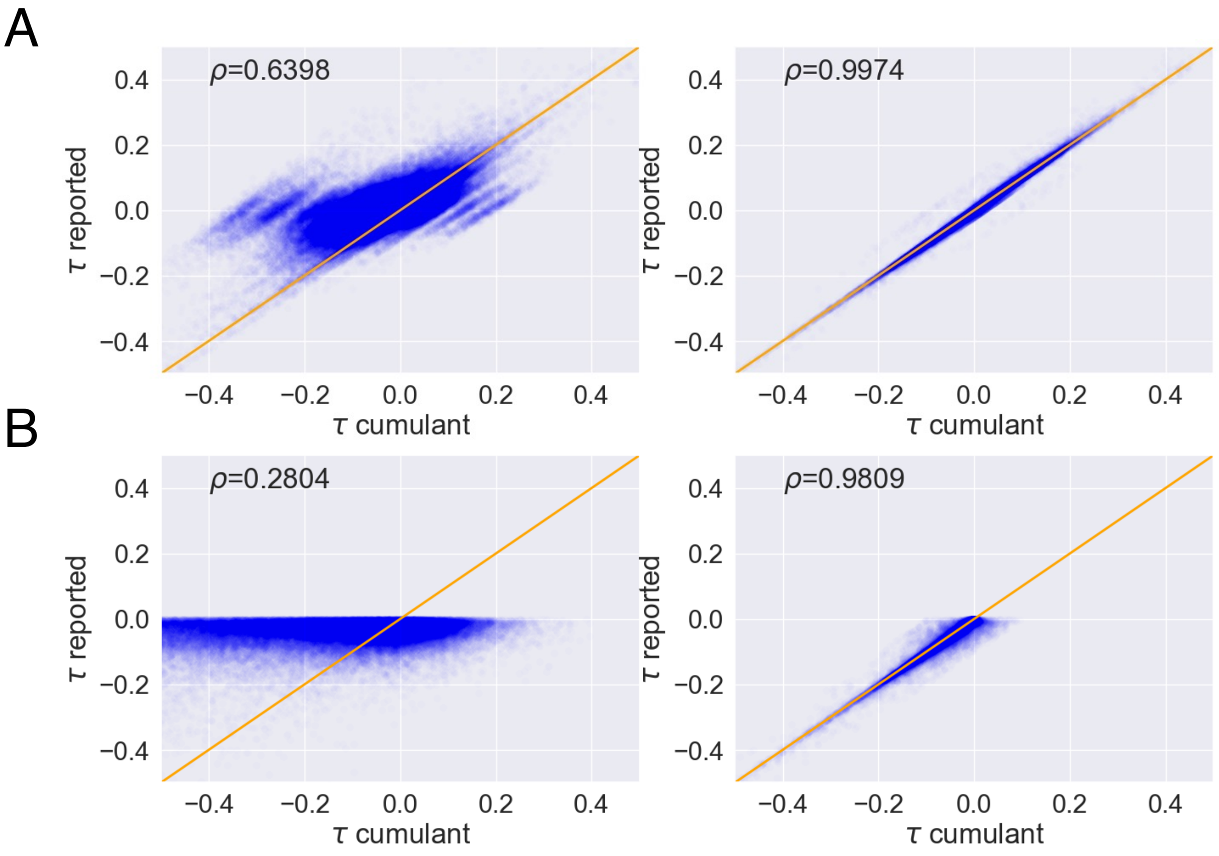

Supplementary Figure 4: **Replication of trigenic interaction scores from Kuzmin et al. [4, 3] calculated using** (19)**. (A-B)** Same analysis as in Figure 3A-B, respectively, except with the trigenic interaction score τ_{ijk} calculated using (19).

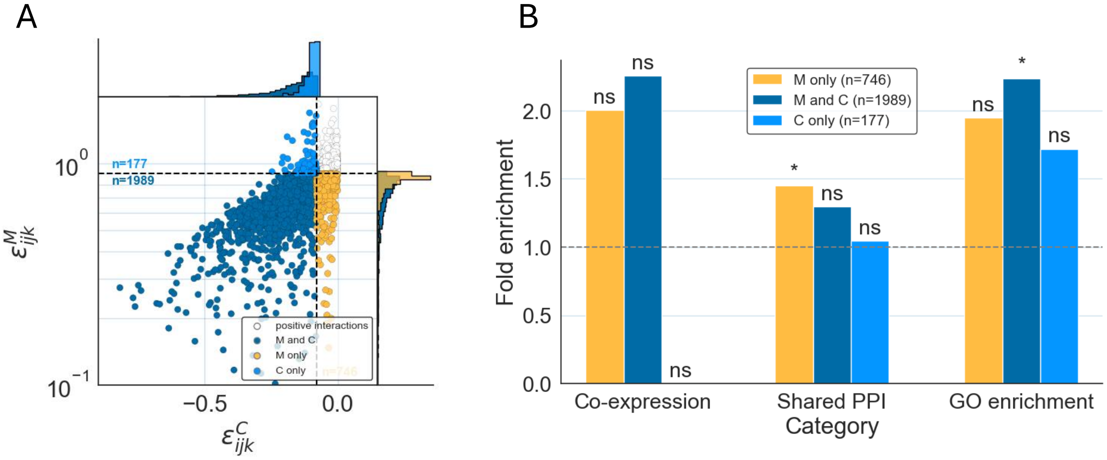

Supplementary Figure 5: **Comparison of negative trigenic interactions reported by Kuzmin et al. (2018) [4] and those detected using the multiplicative epistasis formula. (A-B)** Same analysis as in Figure 4A-B, respectively, but using data from Kuzmin et al. (2018) [4] instead of Kuzmin et al. (2020) [3]. Asterisk (*) denotes statistical significance ($P < 0.022$, hypergeometric test, one-sided), while ‘ns’ indicates not significant ($P > 0.05$).

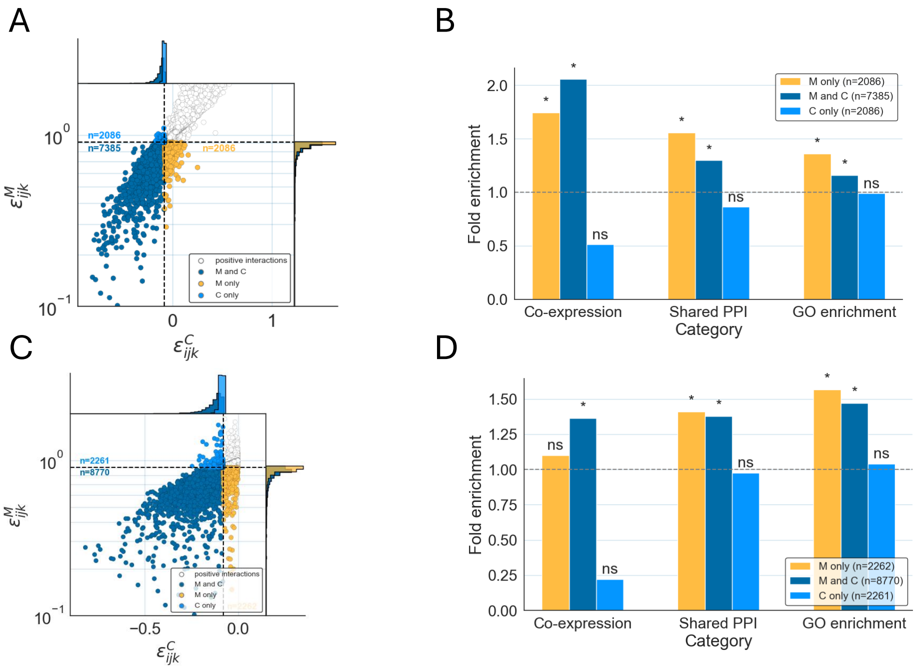

Supplementary Figure 6: **Comparison of negative trigenic interactions reported by Kuzmin et al. (2018) versus those detected using the multiplicative epistasis formula. (A-D)** Comparison of negative trigenic interactions reported by [3] (top row) and [4] (bottom row) and those detected using the multiplicative formula. Panels A-D are identical to the corresponding panels reported in Figure 4 and Figure 5 except we do not use the reported p-value from [4, 3] to filter negative interactions (see Section 2.6). Asterisk (*) denotes statistical significance ($P < 0.045$, hypergeometric test, one-sided), while 'ns' indicates not significant ($P > 0.05$).

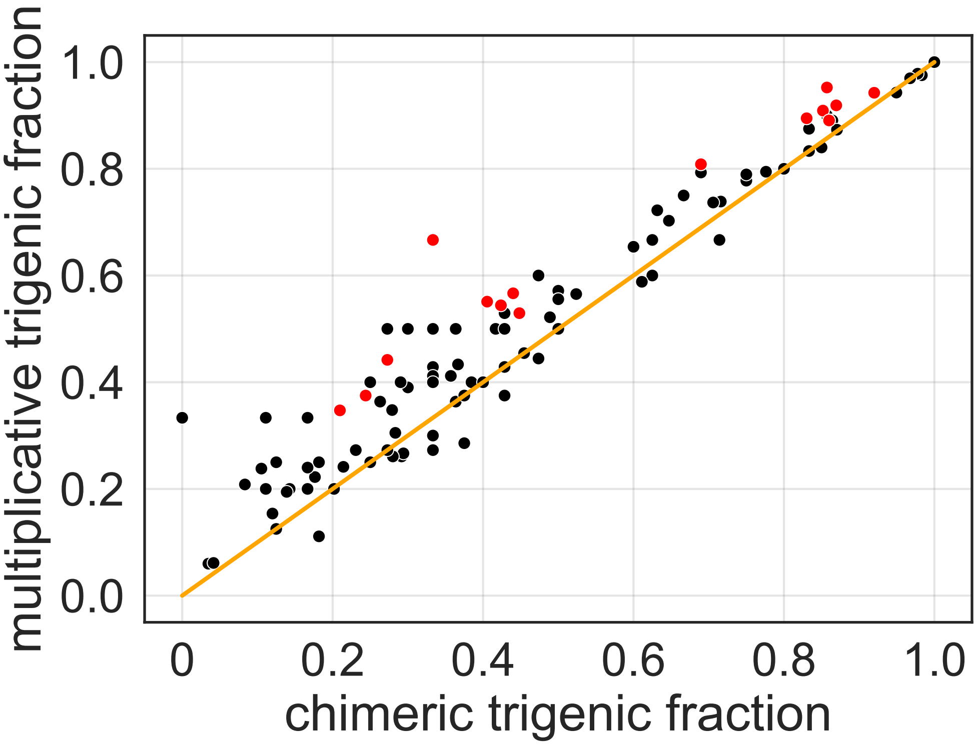

Supplementary Figure 7: **Comparison of trigenic fractions computed using chimeric and multiplicative measures.** Comparison of trigenic fractions computed using the chimeric formula (x-axis) and the multiplicative formula (y-axis) for paralog pairs with at least 6 total interactions (trigenic and digenic). Red dots indicate the paralog pairs highlighted in Figure 4 which had many additional trigenic interactions according to the multiplicative measure. The orange line indicates the point at which trigenic fractions are equivalent across measures.

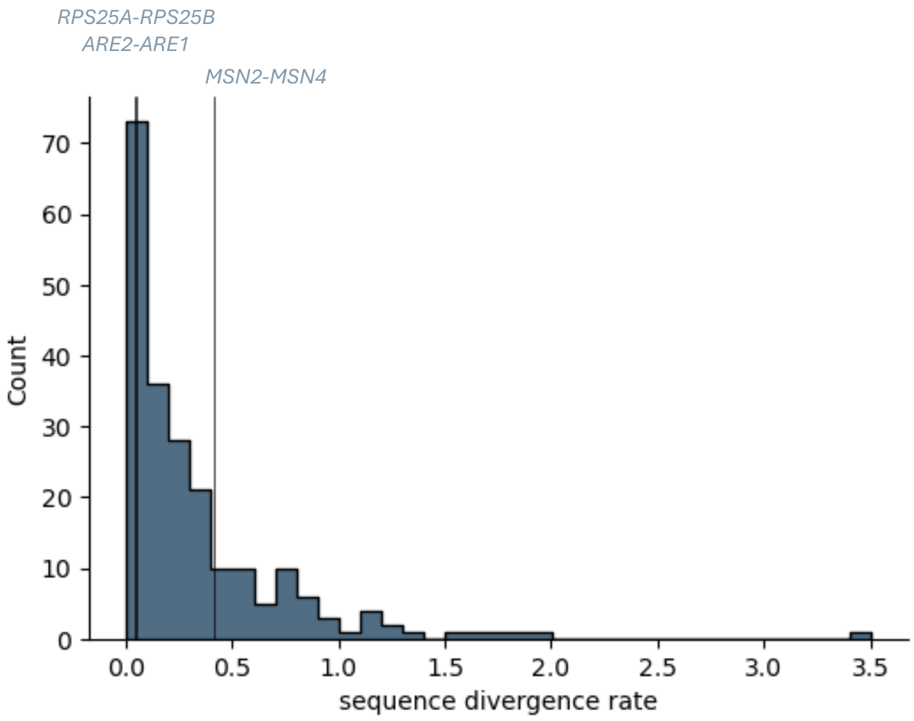

Supplementary Figure 8: **Distribution of sequence divergence rates between paralogs interrogated by [3].** *RPS24A-RPS25B* and *ARE1-ARE2* have low sequence divergence rates of 0.041 and 0.051, respectively, whereas *MSN2-MSN4* has a higher divergence rate of 0.41.

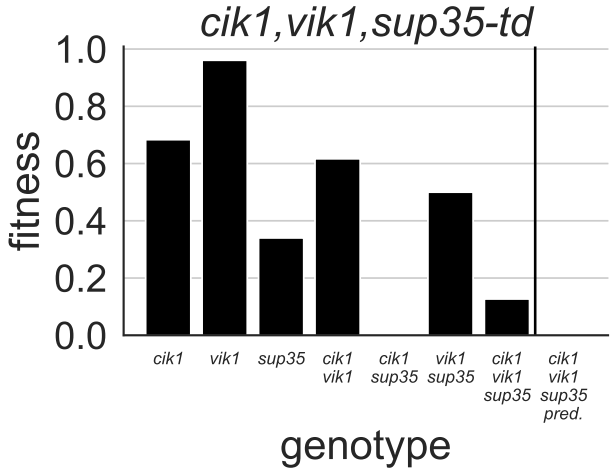

Supplementary Figure 9: **Single, double, and triple mutant fitnesses for the *CIK1*, *VIK1*, *SUP35-td* gene triple.** On the right-hand side of the plot is the predicted triple-knockout fitness using all single mutant fitnesses and pairwise multiplicative epistasis measures ($\epsilon^M_{ij}, \epsilon^M_{ik}, \epsilon^M_{jk}$). The observed triple-knockout fitness is substantially higher than predicted, indicating higher-order epistasis.

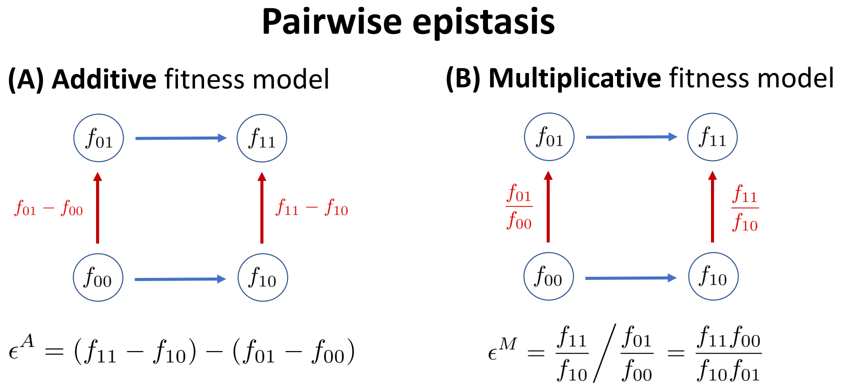

Supplementary Figure 10: **Pairwise epistasis is computed by the change in fitness effect across two possible genetic backgrounds.** Pairwise epistasis is computed by the change in the fitness effect of a mutation in one locus across the two possible genetic backgrounds of the other locus (shown with the two red lines). The computation of the fitness effect and the change in the fitness effect depends on the fitness model. **(A)** In an additive fitness model, the mutational effect is computed with subtraction, while **(B)** in a multiplicative fitness model they are computed with a ratio.

Supplementary References

[1] Frank J Poelwijk, Vinod Krishna, and Rama Ranganathan. The context-dependence of mutations: A linkage of formalisms. *PLoS computational biology*, 12(6):e1004771–e1004771, 06 2016.

[2] Jozef L Teugels. Some representations of the multivariate bernoulli and binomial distributions. *Journal of Multivariate Analysis*, 32(2):256–268, 1990.

[3] Elena Kuzmin, Benjamin VanderSluis, Alex N Nguyen Ba, Wen Wang, Elizabeth N Koch, Matej Usaj, Anton Khmelinskii, Mojca Mattiazzi Usaj, Jolanda Van Leeuwen, Oren Kraus, et al. Exploring whole-genome duplicate gene retention with complex genetic interaction analysis. *Science*, 368(6498):eaaz5667, 2020.

[4] Elena Kuzmin, Benjamin VanderSluis, Wen Wang, Guihong Tan, Raamesh Deshpande, Yiqun Chen, Matej Usaj, Attila Balint, Mojca Mattiazzi Usaj, Jolanda Van Leeuwen, et al. Systematic analysis of complex genetic interactions. *Science*, 360(6386):eaao1729, 2018.

[5] Michael Costanzo, Benjamin VanderSluis, Elizabeth N Koch, Anastasia Baryshnikova, Carles Pons, Guihong Tan, Wen Wang, Matej Usaj, Julia Hanchard, Susan D Lee, et al. A global genetic interaction network maps a wiring diagram of cellular function. *Science*, 353(6306):aaf1420, 2016.

[6] Takeshi Obayashi, Shun Kodate, Himiko Hibara, Yuki Kagaya, and Kengo Kinoshita. COXPRESdb v8: an animal gene coexpression database navigating from a global view to detailed investigations. *Nucleic Acids Research*, pages 1–8, 2022.
