# Supplementary material for: Resolving discrepancies between chimeric and multiplicative measures of higher-order epistasis
Source: Nat Commun. 2025 Feb 17;16:1711. doi: 10.1038/s41467-025-56986-5 (PMC11833126; doi:10.1038/s41467-025-56986-5)
Supplement: Supplementary file 2 — Reporting Summary [file 41467_2025_56986_MOESM2_ESM.pdf]

nature portfolio

Corresponding author(s): Benjamin J. Raphael

Last updated by author(s): Dec 12, 2024

Reporting Summary

Nature Portfolio wishes to improve the reproducibility of the work that we publish. This form provides structure for consistency and transparency in reporting. For further information on Nature Portfolio policies, see our Editorial Policies and the Editorial Policy Checklist.

Statistics

For all statistical analyses, confirm that the following items are present in the figure legend, table legend, main text, or Methods section.

n/a	Confirmed	
☐	☒	The exact sample size (*n*) for each experimental group/condition, given as a discrete number and unit of measurement
☐	☒	A statement on whether measurements were taken from distinct samples or whether the same sample was measured repeatedly
☐	☒	The statistical test(s) used AND whether they are one- or two-sided<br>*Only common tests should be described solely by name; describe more complex techniques in the Methods section.*
☒	☐	A description of all covariates tested
☐	☒	A description of any assumptions or corrections, such as tests of normality and adjustment for multiple comparisons
☐	☒	A full description of the statistical parameters including central tendency (e.g. means) or other basic estimates (e.g. regression coefficient) AND variation (e.g. standard deviation) or associated estimates of uncertainty (e.g. confidence intervals)
☐	☒	For null hypothesis testing, the test statistic (e.g. *F*, *t*, *r*) with confidence intervals, effect sizes, degrees of freedom and *P* value noted<br>*Give P values as exact values whenever suitable.*
☒	☐	For Bayesian analysis, information on the choice of priors and Markov chain Monte Carlo settings
☒	☐	For hierarchical and complex designs, identification of the appropriate level for tests and full reporting of outcomes
☐	☒	Estimates of effect sizes (e.g. Cohen's *d*, Pearson's *r*), indicating how they were calculated

Our web collection on statistics for biologists contains articles on many of the points above.

Software and code

Policy information about availability of computer code

Data collection	There was no data collected in this study.
Data analysis	The data analysis code for this study is provided at https://github.com/raphael-group/chimeric-epistasis/ . It requires the following Python packages:<br>Numpy (>= 1.23.4)<br>Matplotlib (>= 3.8.0)<br>Pandas (>= 2.1.1)<br>Scipy (>= 1.11.2)<br>Seaborn (>= 0.12.2)

For manuscripts utilizing custom algorithms or software that are central to the research but not yet described in published literature, software must be made available to editors and reviewers. We strongly encourage code deposition in a community repository (e.g. GitHub). See the Nature Portfolio guidelines for submitting code & software for further information.

Data

Policy information about availability of data

All manuscripts must include a data availability statement. This statement should provide the following information, where applicable:

- Accession codes, unique identifiers, or web links for publicly available datasets
- A description of any restrictions on data availability
- For clinical datasets or third party data, please ensure that the statement adheres to our policy

The datasets used in this study were obtained through publicly available repositories. The synthetic genetic array (SGA) data used to analyze three-way epistasis in yeast was obtained from https://doi.org/10.5061/ dryad.g79cnp5m9 and https://boonelab.ccbr.utoronto.ca/supplement/kuzmin2018/supplement. html. The drug response data used to analyze higher-order interactions between drug combinations was obtained from the supplementary information of [40]. The deep mutational scanning (DMS) data used to analyze higher-order interactions between protein mutations was obtained from: https://doi.org/10.5281/zenodo.8228919 (FolA); https://github.com/AWHKU/RunMLDE_SpCas9/tree/main (SpCas9); https://github.com/ J-SNACKKB/FLIP/tree/main/splits/gb1 (GB1); https://github.com/ desai-lab/compensatory_ epistasis_omicron/tree/main/Titeseq/results/Kds (Omicron BA.1 variant of SARS-CoV-2); the supplementary information of [80] (eqFP611); http://dx.doi.org/10.6084/m9.figshare.3102154 (avGFP); https://github.com/aequorea238/Orthologous_GFP_Fitness_Peaks (GFPs); the supplementary information of [82] (tRNA); and the supporting information of [83] (CreiLOV). Source data are provided with this paper.

Research involving human participants, their data, or biological material

Policy information about studies with human participants or human data. See also policy information about sex, gender (identity/presentation), and sexual orientation and race, ethnicity and racism.

Reporting on sex and gender	No information on human participants, including on reporting on sex and gender, was collected for this study.
Reporting on race, ethnicity, or other socially relevant groupings	No information on human participants, including on reporting on race, ethnicity, or other socially relevant groupings, was collected for this study.
Population characteristics	No information on human participants was collected for this study, and thus population characteristics are not relevant.
Recruitment	No information on human participants was collected for this study, and thus recruitment is not relevant.
Ethics oversight	No information on human participants was collected for this study, and thus ethics oversight is not relevant.

Note that full information on the approval of the study protocol must also be provided in the manuscript.

Field-specific reporting

Please select the one below that is the best fit for your research. If you are not sure, read the appropriate sections before making your selection.

☒ Life sciences ☐ Behavioural & social sciences ☐ Ecological, evolutionary & environmental sciences

For a reference copy of the document with all sections, see nature.com/documents/nr-reporting-summary-flat.pdf

Life sciences study design

All studies must disclose on these points even when the disclosure is negative.

Sample size	We used publicly available datasets where the sample size, i.e. the number of variants whose fitness was measured, was determined in prior publications. We chose more than 10 datasets to analyze as this number of datasets specifically demonstrates our findings on yeast, drug interactions, and proteins.
Data exclusions	We did not exclude any data from our analyses.
Replication	We analyzed more than 10 publicly available datasets in order to demonstrate our findings robustly.
Randomization	We use publicly available data so we do not need to randomize any samples.
Blinding	We did not collect any data and used publicly available data, and thus did not need to use blinding.

Reporting for specific materials, systems and methods

We require information from authors about some types of materials, experimental systems and methods used in many studies. Here, indicate whether each material, system or method listed is relevant to your study. If you are not sure if a list item applies to your research, read the appropriate section before selecting a response.

Materials & experimental systems

n/a	Involved in the study
☒	☐ Antibodies
☒	☐ Eukaryotic cell lines
☒	☐ Palaeontology and archaeology
☒	☐ Animals and other organisms
☒	☐ Clinical data
☒	☐ Dual use research of concern
☒	☐ Plants

Methods

n/a	Involved in the study
☒	☐ ChIP-seq
☒	☐ Flow cytometry
☒	☐ MRI-based neuroimaging

Plants

Seed stocks

We did not study plants and thus did not use seed stocks, so this is not relevant to our study.

Novel plant genotypes

We did not study plants or analyze plant genotypes, and thus this question is not relevant to our study.

Authentication

We did not study plants or need to validate seed stocks, and thus this question is not relevant to our study.
